# Book Reviews

**Published:** 1897-06

**Authors:** 


### BOOK REVIEWS.

DISEASES OF THE EAR, NOSE AND THROAT AND THEIR ACCESSORY CAVITIES. A Condensed Text-book. By Seth Scott Bishop, M.D., LL.D., Professor in the Chicago Post-Graduate Medical School and Hospital; Surgeon to the Illinois Charitable Eye and Ear Infirmary; etc. Illustrated with 100 colored lithographs and 168 additional illustrations. One volume, royal octavo, pages xvi–496. Extra cloth, $4.00. The F. A. Davis Co., Publishers, 1914 and 1916 Cherry Street, Philadephia.

"This work was designed, first, for students, and, second, for those progressive practitioners who wish to acquire the proficiency necessary to properly treat those patients who are unable to visit specialists; and, third, for those who are gradually exchanging their general practice for special work in these branches. The subjects are so simplified and condensed as to form an introduction to the exhaustive treatises already in the field."

The book very well sustains the author's purpose. He has presented the diseases of the ear, nose and throat in their important and essential features, omitting unsettled and disputed questions. Very properly he devotes little space to the anatomy of the various organs; it is assumed that the reader either knows this or can obtain it elsewhere. Thus full space is reserved for the description of diseases and their treatment. One hundred and eighty-eight pages are devoted to the ear, in which the author follows the usual classification of diseases. The nose gets one hundred and fifteen pages. In this section the chapters on hay fever are particularly full and interesting. Diseases of the pharynx embrace one hundred and ten pages, and those of the larynx seventy pages. The colored lithographs are true to life and are well executed.

While the book is by no means an exhaustive treatise, it is a safe and practical guide to the study of diseases of these organs, and we take pleasure in recommending it as a very successful effort in its field.

SYRINGOMYELIA. An Essay to which was Awarded the Alvarenga Prize of the College of Physicians of Philadelphia for the year 1895. By Guy Hinsdale, A.M., M.D., Fellow of the College of Physicians of Philadelphia, etc., etc., etc. Published by P. Blakiston, Son & Co., Philadelphia. Price $1.00.

The world of books constantly appearing gives the reviewer the onerous task of selecting or approving little out of much. Few text-books, collaborated treatises, or monographs contain much that is new; but accepting the oriental maxim, that "all printed matter contains something instructive," we can give time and effort to ceaseless study, being assured that the gleaning may prove valuable.

Dr. Hinsdale, judging from the extensive bibliography annexed, has given much time and deep study to the subject, Syringomyelia. The author's style is pleasing, the illustrated cases most instructive and typical. The diagrams, though not exquisite, are graphic. Above all, the essay is, so far as my humble judgment goes, the best and most exhaustive I have seen to date.

It were an easy task, having just enjoyed the rich store of information contained within these seventy pages, to give vent to great praise; but I will forbear, only saying that in Dr. Hinsdale's Essay the profession has a gem, and the neurologist and general practitioner will find, that after careful study of this book, he will be much better capacitated to diagnosticate this rare, complex and interesting disease.

The volume is a neat, well printed one. H. H.

---

RETINOSCOPY. By James Thorington, M.D., Adjunct Professor of Diseases of the Eye in the Philadelphia Polyclinic and College for Graduates in Medicine, etc., etc. Twenty-four illustrations. Price $1. P. Blakiston, Son & Co., 1012 Walnut St., Philadelphia.

In this little book the author endeavors to describe the application of the shadow-test in the simplest manner possible; therefore he considers only the use of the *plane mirror at one meter distance.* He is an earnest advocate of this method of estimating the error of refraction, and in the beginning he lays down this axiom : "*With an eye otherwise normal, except for its refractive error, and being under the influence of a reliable cyclopegic, there is no more accurate objective method of obtaining its exact correction than by retinoscopy.*" The author describes the practical use of retinoscopy with the plane mirror, the point of reversal, the arrangement of patient, light and observer, the direction of movement of the retinal illumination, the rules for correcting lenses in the various forms of ametropia, etc.

Any one who desires to study this method and to familiarize himself with this additional means of measuring refractive errors will find this book very helpful.

A PRACTICAL TREATISE ON DISEASES OF THE SKIN, FOR THE USE OF STUDENTS AND PRACTITIONERS. Fourth and Revised Edition. By James Nevins Hyde, A.M., M.D., Professor of Skin and Venereal Diseases, Rush Medical College, Chicago; Dermatologist to the Presbyterian and Michael Reese Hospitals of Chicago, etc., etc., and Frank H. Montgomery, M.D., Lecturer on Dermatology and Genito-Urinary Diseases, and Chief Assistant to the Clinic for Skin and Venereal Diseases, Rush Medical College, Chicago, etc. Illustrated with one hundred and ten engravings and twelve plates in colors and monochrome. Lea Bros. & Co., Philadelphia and New York.

The preceding edition of this work was reviewed in THE JOURNAL. Hyde's is one of the best works on Diseases of the Skin, of which there are many. Much new matter, many descriptions of new diseases and additional text have been added in this last edition.

The text is well written and the illustrations are good.

A critique of such a well-known work would be out of place here. Suffice it to say, the book is a standard, and one would be safe in using it as a guide.

The enormous number of diseases of the skin which have different names, but are much alike, as shown in this and other text-books, makes us long for the time when there will be a contraction of terminology, and a reduction in the number of distinctly named diseases. Yet, with all the fulness of the book, there is the omission of at least one distinct disease; Mibelli's Porokeratosis, or Respighii's Hyperkeratosis Excentrica. M. B. H.

---

LEPROSY AND THE CHARITY OF THE CHURCH. By Rev. L. W. Mulhane, Mt. Vernon, Ohio. D. H. McBride & Co., Chicago and New York. Price, 75c.

This is a little book which gives the history of leprosy in ancient times, then in the middle ages, and finally at the present time. There is a description of leprosy as found in the United States, and a considerable amount of space is devoted to the dangers from the disease to the people of this country. He has a general chapter upon the nature and treatment of the disease, and quotes many "authorities."

The latter part of the book is devoted to what "The Church" has done for these unfortunates.

This little work is quite interesting but, obviously, more so to the laity than it can be to physicians who may feel special interest in *studying* the subject. M. B. H.

